# Dental-derived Stem Cells and whole Tooth Regeneration: an Overview

**DOI:** 10.4021/jocmr2009.03.1230

**Published:** 2009-03-31

**Authors:** Aous Dannan

**Affiliations:** Department of Periodontology, Faculty of Dental Medicine, Witten/Herdecke University, Witten, Germany. Email: aousdannan@yahoo.com

## Abstract

The need for new dental tissue-replacement therapies is evident in recent reports which reveal startling statistics regarding the high incidence of tooth decay and tooth loss. Recent advances in the identification and characterization of dental stem cells, and in dental tissue-engineering strategies, suggest that bioengineering approaches may successfully be used to regenerate dental tissues and whole teeth. Interest in dental tissue-regeneration applications continues to increase as clinically relevant methods for the generation of bioengineered dental tissues, and whole teeth, continue to improve. This paper is concerned about dental-derived stem cells and their characterization. Additionally, since conventional dental treatments partially serve the purpose for replacing missing teeth and always include possible failure rates, the potential of dental-derived stem cells in promoting whole tooth regeneration is also discussed.

**Keywords:**

Dental stem cells; Tissue engineering; Tooth regeneration

## Introduction

Teeth are structures that develop on the maxilla and mandible of mammals and serve eating, defense and phonetic purposes. Although the morphology of teeth varies depending on species and location, they are similar in structure, being composed of enamel, dentin, pulp and periodontium. Tooth development is characterized by a series of reciprocal epithelial mesenchymal interactions that result in differentiation and the spatial organization of cells to form organs [1, 2]. Since gene expression comparisons during teeth development have shown only slight differences between human and mouse teeth, mice have been used as the major animal model for studying tooth development. Human genetic diseases that encompass loss of teeth also contribute to the understanding of tooth formation [3, 4].

Modern dentistry for replacing missing teeth utilizes metal implants capped with a ceramic crown [5]. Although these prostheses serve the purpose, factors that interfere with osseointegration may cause surgery failure [6]. With advances in stem cell biology and emerging concepts of tissue engineering [7], biological teeth [8] may become an alternative for replacing missing teeth. The idea is to cultivate stem cells with odontogenic induction signals through epithelial-mesenchymal interactions, thereby programming the stem cells to adopt dental lineages and, with the help of scaffold matrix, to become part of a tooth.

## Tooth development: an ideal model for tooth regeneration

In order to understand how tooth regeneration should be induced, a deep knowledge of the normal tooth development is always a prerequisite.

Generally, tooth development is initiated by the dental epithelium and proceeds through five distinct morphological stages: bud, cap, bell, crown, and root [2, 9]. The coordinated development of tooth supporting structures, including periodontal ligament (PDL) and alveolar bone, begins around the bell stage. The tooth germ is first identifiable as a localized thickening and proliferation of the oral epithelium. The dental epithelium forms a bud that extends into the underlying dental mesenchyme, marking the first stage of tooth development. The dental epithelium subsequently undergoes significant proliferative activity, extending around the periphery to form a cap-like structure.

During this process, the non-proliferating enamel knot signaling center [10] becomes identifiable, as epithelial cells organize themselves into three distinct regions, namely the outer epithelium, the inner epithelium, and central cell layers called the *stratum intermedium* and *stellate reticulum*. The ectomesenchymal cells of the dental papilla condense beneath the invaginating dental epithelium, eventually giving rise to dentin and pulp tissues. The dental follicle forms around the enamel organ and dental papilla, eventually forming the periodontal tissues.

The bell stage is characterized by continued proliferation and histo-differentiation of the dental epithelium. Inner dental epithelial cells assume a cuboidal shape and produce high levels of glycogen, adjacent *stratum intermedium* produces high levels of alkaline phosphatase, and the *stellate reticulum* assumes a distinctive star shape, surrounded by the outer epithelial cell layer.

As tooth development proceeds through differentiation stages, dental mesenchyme-derived odontoblasts differentiate and elaborate the dentin matrix, and epithelial cell-derived ameloblasts cells secrete the enamel matrix for enamel production. After the tooth crown has formed, tooth root structures develop from the rudimentary Hertwig's epithelial root sheath, forming dentin, cementum, periodontal ligament and alveolar bone.

In naturally formed teeth, enamel - the only mineralized tooth tissue derived from the dental epithelium - exhibits no regenerative properties, while the remaining mineralized periodontal and dental tissues, including dentin, pulp, cementum, periodontal ligament and alveolar bone, all of which are formed from neural crest-derived dental ectomesenchyme, each exhibit a certain degree of regenerative capability which is supposed to be, partially, related to the existence of stem cells.

## Stem cells

Stem cells are one of the most fascinating areas of biology today. But like many expanding fields of scientific inquiry, research on stem cells raises scientific questions as rapidly as it generates new discoveries.

Stem cells differ from other kinds of cells in the body. All stem cells, regardless of their source, have three general properties: they are capable of dividing and renewing themselves for long periods; they are unspecialized; and they can give rise to specialized cell types [11].

Unlike muscle cells, blood cells, or nerve cells -which do not normally replicate themselves- stem cells may replicate many times. When cells replicate themselves many times over it is called proliferation. A starting population of stem cells that proliferates for many months in the laboratory can yield millions of cells. If the resulting cells continue to be unspecialized, like the parent stem cells, the cells are said to be capable of long-term self-renewal.

Totipotent stem cells are produced from the fusion of an egg and sperm cell. Cells produced by the first few divisions of the fertilized egg are also totipotent. These cells can differentiate into embryonic and extra embryonic cell types.

Pluripotent stem cells are the descendants of totipotent cells and can differentiate into cells derived from any of the three germ layers.

Multipotent stem cells can produce only cells of a closely related family of cells (e.g. hematopoietic stem cells differentiate into red blood cells, white blood cells, platelets, etc.).

Unipotent cells can produce only one cell type, but have the property of self-renewal which distinguishes them from non-stem cells (e.g. muscle stem cells).

Properties of stem cells can be illustrated in vitro, using methods such as clonogenic assays, where single cells are characterized by their ability to differentiate and self-renew [12, 13]. As well, stem cells can be isolated based on a distinctive set of cell surface markers. However, in vitro culture conditions can alter the behavior of cells, making it unclear whether the cells will behave in a similar manner in vivo. Considerable debate exists whether some proposed adult cell populations are truly stem cells.

In dental research, there are two major categories of stem cells which are discussed for clinical application: embryonic stem cells and somatic (or adult) stem cells. Isolation and use of human embryonic stem cells is ethically controversial, but first evaluations are on the way for actual treatments [14].

Somatic stem cells have a limitation in their potentials of differentiation. However, it is thought that somatic or adult stem cells are a better option for dentistry, as these cells are easily accessible, and their use does not bring up ethical concerns [15].

## Location of stem cells within dental tissues

Recently, it has been found that specific populations of stem cells and/or progenitor cells could be isolated from three main dental resources, namely the dental follicle, the dental pulp and the developed periodontal ligament.

### Stem cells in the dental follicle

One important biological function of the dental follicle is the coordination of tooth eruption [16]. Moreover this tissue harbors progenitor cells for the periodontal ligament [17]. The differentiation and function of dental follicle cells are controlled by a network of regulatory molecules including growth factors and cytokines. It is thought that the dental follicle cells near the forming root (innermost) differentiate into cementum forming cementoblasts and that cells towards the alveolar bone (outermost) differentiate to osteoblasts secreting bone matrix. Dental follicle cells found centrally between the cementoblast and osteoblast precursor cells develop into fibroblasts producing the extracellular matrix of the periodontal ligament.

Mechanisms, which regulate follicle cell differentiation, are, in the beginning, to be understood. Cementogenesis during tooth development is dependent on root formation. Root formation begins by proliferation of the outer and inner enamel epithelium forming the bilayered Hertwig's epithelial sheath. Epithelial stimuli derived from this root sheath seem to be responsible for the differentiation of follicle precursor cells into cementoblasts [18, 19].

It was demonstrated that Bone Morphogenetic Protein-2 (BMP-2) promotes differentiation of immortalized dental follicle cells towards an osteoblast and/or cementoblast phenotype [20]. Moreover, dental follicle progenitor cells were isolated from bovine tooth germs by digestion with bacterial collagenase [21, 22]. The differentiation capacity of these cells was proved by in vivo tests with mice. Here, cells formed a cementum-like matrix in contrast to bovine PDL fibroblasts or bovine alveolar osteoblasts. In contrast to in vivo tests, no differentiation potential was observed in long term cultures in the presence of dexamethasone.

Later after, Morsczeck and co-workers [23] reported the isolation of precursor cells derived from dental follicle of human third molar teeth. These fibroblast-like, colony-forming and plastic adherent cells expressed putative stem cell markers; Notch-1 and Nestin. It has also been shown that long-term cultures with dexamethasone produced compact calcified nodules or appeared as plain membrane structures of different dimensions consisting of a connective tissue like matrix encapsulated by a mesothelium-like cellular structure. Therefore, these results demonstrated that cultured precursor cells are unique undifferentiated lineage committed cells residing in the periodontium prior or during tooth eruption. In a recent study, it was hypothesized that stem cells may be present in the dental follicle and be capable of differentiating into cells of the periodontium [24]. At the same field of another study, it was demonstrated that human third molar pad possesses neural crest-derived cells that represent multipotent stem/progenitor cells [25]. For future studies, however, the dental follicle's stem cells will need to be further characterized.

### Stem cells in the dental pulp

The dental pulp is the part in the center of a tooth made up of living soft tissue and cells called odontoblasts. The central region of the coronal and radicular pulp contains large nerve trunks and blood vessels. This area is lined peripherally by a specialized odontogenic area which has three layers which are (from innermost to outermost): cell rich zone, cell free zone and odontoblastic layer.

During tooth formation, interactions between epithelial and dental papilla cells promote tooth morphogenesis by stimulating a subpopulation of mesenchymal cells to differentiate into odontoblasts, which in turn form primary dentin. These odontoblasts are thought to arise from the proliferation and differentiation of a precursor population, residing somewhere within the pulp tissue [26].

It is known that in certain conditions, cultures of pulp cells derived from early developing dental root tissue and pulp tissue can develop an odontoblast-like appearance with the capacity to form mineralized nodules in vitro [27], a trait normally attributed to cultures of bone or bone marrow cells [28, 29].

It has been speculated that adult dental pulp tissue might also contain a population of multipotential stem cells [30], and that postnatal dental pulp contains cells that are clonogenic, highly proliferative, and capable of regenerating a tissue, properties that effectively define them as stem cells. It has also been shown that Dental Pulp Stem Cells (DPSCs) were capable of forming ectopic dentin and associated pulp tissue in vivo [31]. Stromal-like cells were re-established in culture from primary DPSCs transplants and retransplanted into immunocompromised mice to generate a dentin-pulp-like tissue, demonstrating their self renewal capability [31].

Stem cell cultures have been successfully established from both dental pulp tissue and periodontal ligament by Varga and co-workers [32] who demonstrated that both cell cultures showed typical fibroblast-like morphology, showing clonogenic activity. STRO-1 immunoreactivity in both cell cultures was also detected.

Taken together, these results demonstrated that DPSCs possess stem-cell-like qualities, including self-renewal capability and multi-lineage differentiation.

Another study has provided evidence that DPSCs and their osteoblast differentiated cells can be safely recovered after long-term cryopreservation [33]. All these features and abilities make these cells attractive for therapeutic 3-dimensional tissue reconstruction, with the potential of tailoring storage and recovery to the needs of the patient.

In the same field, it has been found that exfoliated human deciduous tooth contains multipotent stem cells [Stem cells from Human Exfoliated Deciduous teeth (SHED)] [34]. SHED were identified to be a population of highly proliferative, clonogenic cells capable of differentiating into a variety of cell types including neural cells, adipocytes, and odontoblasts. After in vivo transplantation, SHED were found to be able to induce bone formation, generate dentin, and survive in mouse brain along with expression of neural markers. SHED are not only derived from a very accessible tissue resource but are also capable of providing enough cells for potential clinical application. Thus, it has been shown that exfoliated teeth may be an unexpected unique resource for stem-cell therapies, including autologous stem-cell transplantation and tissue engineering.

In a trial to model the epithelial-mesenchymal interactions which take place during tooth germ differentiation and to focus on the importance of DPSCs at this level, Kadar and co-workers [35] used cultures of mesenchymal cells isolated from adult human dental pulp and undifferentiated epithelial cells from salivary origin, and it has been shown that coculturing of DPSCs and epithelial cells resulted in dramatic changes of cell shape and migrating activity of DPSCs, while no obvious morphological change can be observed in the epithelial cells.

Szlavik and co-workers tried to characterize the neuronal differentiation in a culture of DPSCs and SHED, and it was shown that induction of neuronal differentiation can be achieved by retinoic acid in the pulp originated cultures. When cultured together with undifferentiated epithelial cells, DPSC and SHED cells showed similar signs of neuronal differentiation suggesting the high plasticity and transdifferentiation potency of these cell populations [36].

Moreover, it has been suggested that functional evidence for neuronal differentiation of human DPSCs in vitro may exist [37].

### Stem cells in the periodontium

The periodontal ligament is a group of specialized connective tissue fibers that essentially attach a tooth to the alveolar bone within which it sits. These fibers help the tooth withstand the naturally substantial compressive forces which occur during chewing and remain embedded in the bone. Another function of the PDL is to serve as a source of proprioception, or sensory innervations, so that the brain can detect the forces being placed on the teeth and react accordingly. In addition to the PDL fibers, there is another set of fibers, known as the gingival fibers, which attach the teeth to their adjacent gingival tissue. Both the gingival fibers, as well as the PDL fibers, are composed primarily of type I collagen.

The periodontal ligament is derived embryologically from the ectomesenchymal tissue of the dental follicle that surrounds the developing tooth in its bony crypt. At the time of tooth eruption the cells and collagen fibers in the dental follicle, i.e. the future periodontal ligament, are orientated primarily with their long axis parallel to the root surface. Remodeling of the follicle into a periodontal ligament begins at the cemento-enamel junction and proceeds in an apical direction.

The periodontal ligament contains a unique assortment of cells that are capable of generating and maintaining three distinct tissues, namely the ligament itself as well as the mineralized tissues on either side of it, i.e. the cementum and the alveolar bone. The major cell types of the periodontal ligament include: Fibroblasts, macrophages and undifferentiated ectomesenchymal cells, cementoblasts and cementoclasts, osteoblasts and osteoclasts, cell rests of Malassez, vascular and neural elements.

PDL contains heterogeneous cell populations [38, 39] that can differentiate into either cementum-forming cells (cementoblasts) or bone-forming cells (osteoblasts) [40-43]. Recent findings suggest that PDL cells have many osteoblast-like properties, including the capacity to form mineralized nodules in vitro, expression of the bone-associated markers alkaline phosphatase and bone sialoprotein, and response to bone-inductive factors such as parathyroid hormone, insulin-like growth factor 1, bone morphogenetic protein 2 and transforming growth factor β1 [44-49]. The presence of multiple cell types within PDL has led to speculation that this tissue might contain progenitor cells that maintain tissue homoeostasis and regeneration of periodontal tissue [40, 50-52].

Using a methodology similar to that utilized to isolate Mesenchymal Stem Cells (MSCs) from deciduous and adult dental pulp, multipotent postnatal stem cells from human periodontal ligament or Periodontal Ligament Stem Cells (PDLSCs) have also been isolated and described [32, 53-63]. Cultured cells were expanded from single cell suspensions derived from periodontal ligament tissue and the presence of stem cells was determined using antibodies such as STRO-1 and CD146. Under defined cultured conditions, PDLSCs were able to differentiate into cementoblast-like cells, adipocytes and collagen-forming cells.

Immunohistochemical staining and Western blot analysis showed that cultured PDLSCs express an array of cementoblastic/osteoblastic markers, including alkaline phosphatase, bone sialoprotein, osteocalcin and transforming growth factor-β receptor type I. These PDLSCs were transplanted into artificially created periodontal defects in the mandibular molars in rats. Histological evaluations 68 weeks after implantation showed that these cells had the capacity to generate a thin layer of cementum-like tissue on the surface of the hydroxyapatite/tricalcium phosphate ceramic particles carrier, along with condensed collagen fibers that resembled Sharpey's fibers [61].

The presence of MSCs in the periodontal ligament is also supported by other findings where a population of MSCs from the periodontal ligament has been isolated and characterized showing the ability to express a variety of stromal cells markers (CD90, CD29, CD44, CD166, CD105, CD13) [60, 64]. The clinical potential for the use of PDLSCs has been further enhanced by the demonstration that these cells can be isolated from cryopreserved periodontal ligaments, thus providing a ready source of MSCs [62].

Moreover, putative stem cells in both healthy and diseased periodontal ligament could be identified [53]. They were mainly located in the paravascular region and small clusters of cells were also found in the extra-vascular region. Wider distributions of these cells were detected in sections of diseased ligament.

In a recent study, our research group described a method for serum-free culture, rapid expansion and subsequent efficient induction of neuronal phenotype in the so-called periodontium-derived neural stem cells (PdNSCs) [65]. The isolated, cultured, highly proliferative cells were positive for the neural stemness markers Nestin and Sox2. In addition, the PdNSCs migrated when exposed to chemokines that are reported to be migration-inducing in neural stem cells isolated from subventicular zone. Retinoic acid treatment efficiently induced neuronal fate in PdNSCs as shown by the high levels (> 90%) of neuron-specific markers. However, the capacity of these periodontium-derived stem cells to function in vivo remains unresolved.

More recently, many studies have investigated the behavior of stem cells in the periodontium during wound healing of bone defects [66], and their involvement in periodontal regeneration [67]. These studies showed that cells with characteristics of putative mesenchymal stem cells were found in regenerating periodontal tissues, implying their involvement in periodontal regeneration. Moreover, mesenchymal stem cells and hematopoietic stem cells in the bone marrow were not involved in the regeneration of the periodontium. Cells that migrated from the residual periodontal ligament regenerated new alveolar bone at early stages.

It could be stated that within the periodontal ligament of both healthy and diseased teeth, cells have been identified consistent with their identification as putative stem cells [68-70]. The presence of an inflammatory reaction associated with periodontitis may enhance the number of these cells, and their role in periodontal tissue regeneration might be of great importance as a step on the road of dental tissue regeneration.

### Whole tooth regeneration

As bioengineered tooth crown formation requires the interactions of both dental epithelial cell progenitors and mesenchymal cell progenitors (as in natural tooth formation), the ability to bioengineer a tooth of specified size and shape will depend on the ability to first identify, and then guide, the interactions of both types of cells. Methods to guide the interactions of epithelial and mesenchymal postnatal dental stem cells to form dentin and enamel layers characteristic of natural teeth, using modified scaffold materials and designs, for example, must be developed. The importance of scaffold materials and design for tissue engineering has long been recognized. Scaffold porosity, biocompatibility and biodegradability, the ability to support cell growth, and use as a controlled gene- and protein-delivery vehicle [71] are all highly significant properties. A variety of hydrophilic polymers have been synthesized that provide cell support and guidance. Importantly, scaffold materials provide a three-dimensional macromolecular structure to guide the final shape of bioengineered tissues. Poly-L-lactic acid and poly lactic co-glycolic acid co-polymers have been used to generate composite scaffolds that degrade within a period of a few weeks up to 1 year [72]. Poly-Lactic acid sponges can support the growth of chondrocytes in a uniform cellular distribution, their utility has been demonstrated in cartilage tissue regeneration [72], and polyglycolic acid and polylactic acid have been shown to support the growth of biopsied neonatal intestine cells into functional, small intestinal tissue [73].

Optimized polymer fabrication techniques have been used to generate three-dimensional structures composed of an intercommunicating network of pores, where the resulting morphology and mechanical properties of the scaffold walls were found to influence tissue engineering applications. However, each type of scaffold has unique features that provide flexibility for a variety of tissue-engineering applications.

From early studies, we know it is possible to regenerate tooth crowns if suitable environments are provided, such as in vitro organ culture, grafts on chick chorio-allantoic membrane, ocular grafts, subcutaneous transplants or kidney capsules [74-78]. These culture sites provide nutrients and oxygen to nurture tooth germs. Thus, several choices exist for cultivating small-sized primordia, such as those of teeth, before they can be implanted into their anatomical sites.

Optimally, the setting should reproduce cells in a three dimensional organization, support the differentiating function, and avoid xenograft rejection.

According to Yen and Sharpe [79], two current ways of tooth tissue engineering are now thinkable. The first method includes growing of dissociated tooth germs on a tooth-shaped scaffold and produce small complex tooth-like structures. The second method includes growing of epithelial and mesenchymal (stem) cells, either from tooth germs or other sources, in organ culture and, through epithelial-mesenchymal interactions, forming organized teeth. In our opinion, however, the first method seems to be more plausible since its elements are more controllable.

Considering the regeneration of a functional and living tooth, Sonoyama and co-workers conducted an interesting study to explore the potential for reconstructing a functional tooth in miniature pigs (mini-pigs) [80], in which a bio-root periodontal complex is built up by postnatal stem cells including stem cells from root apical papilla (SCAP) and PDLSCs, to which an artificial porcelain crown is affixed.

It has been shown that the root apical papilla contained mesenchymal stem cells that appear to have a greater capacity for dentin regeneration than DPSCs. These findings suggest that developing tissue may contain a good stem cell resource for tissue regeneration. SCAP represent a novel population of multipotent stem cells as demonstrated by their capacity to develop into odontoblast-like cells and adipocytes in vitro. This cell population was found to express high levels of survivin and telomerase, which are both important molecules in mediating cell proliferation. In addition, CD24, marker for undifferentiated SCAP, which is downregulated following odontogenic differentiation. These data support the notion that SCAP are a unique population of postnatal stem cells.

Although dental pulp contains DPSCs with dentin/pulp regenerative capacity, developing tissue-derived SCAP showed a superior tissue regeneration potential than that of DPSCs. SCAP collected from just one tooth are capable of providing a large number of stem cells probably sufficient for human transplantation because they have high proliferative potential, reflected in high telomerase activity [81].

Although newly formed bio-roots show a lower compressive strength than that of natural swine root dentin, they seemed capable of supporting porcelain crown and resulted in normal functions. It may be possible to improve the compressive strength and hardness of the bio-roots by selecting optimal bioengineered materials and by optimizing the implanted stem cell numbers and quality.

This hybrid strategy of autologous dental stem cell engineering may be applicable to human tooth regeneration. Furthermore, functional tooth restoration in swine may shed light on human tooth regeneration in the future because of the close similarities between swine and human dental tissues.

We believe that the model of Sonoyama and co-workers provided a unique imagination of how to use dental stem cells to create a functional tooth, which is, practically, the most important unit in replacing missed teeth. However, in order to optimize such models, further investigations should be carried in order to test the stem cells-scaffold interrelationships.

## Conclusions

Complex human tissues harbor stem cells and/or precursor cells, which are responsible for tissue development and repair. Recently, dental tissues such as periodontal ligament, dental papilla or dental follicle have been identified as easily accessible sources of undifferentiated cells.

Dental stem cells have been isolated according to their anatomical locations, colony-forming ability, expression of stem cell markers, and regeneration of pulp/dentin and/or periodontium/cement structures in vivo. These dental-derived stem cells are currently under increasing investigation as sources for tooth regeneration and repair.

Although many challenges remain, stem-cell-based tissue engineering of teeth could be a choice for the replacement of missing teeth in the future.
